# Caregivers' burden and deep brain stimulation for Parkinson disease: A systematic review of qualitative studies

**DOI:** 10.1111/ene.16149

**Published:** 2023-11-17

**Authors:** Francesco Cavallieri, Luca Ghirotto, Francesca Sireci, Margherita Parmeggiani, Cristina Pedroni, Felipe Andres Mardones, Maria Chiara Bassi, Valentina Fioravanti, Valérie Fraix, Elena Moro, Franco Valzania

**Affiliations:** ^1^ Neurology Unit, Neuromotor and Rehabilitation Department Azienda USL–IRCCS di Reggio Emilia Reggio Emilia Italy; ^2^ Qualitative Research Unit Azienda USL–IRCCS di Reggio Emilia Reggio Emilia Italy; ^3^ Direzione delle Professioni Sanitarie Azienda USL–IRCCS di Reggio Emilia Reggio Emilia Italy; ^4^ Servizio di Emergenza Territoriale Modena Italy; ^5^ Medical Library Azienda USL–IRCCS di Reggio Emilia Reggio Emilia Italy; ^6^ Division of Neurology, Centre Hospitalier Universitaire de Grenoble, Grenoble Institute of Neuroscience Grenoble Alpes University Grenoble France

**Keywords:** burden, care partner, caregiver, deep brain stimulation, meta‐synthesis, Parkinson's disease, qualitative research

## Abstract

**Background and purpose:**

The impact of subthalamic nucleus deep brain stimulation (STN‐DBS) on caregivers' burden is understudied. We perform a systematic review and meta‐synthesis aggregating qualitative studies involving partners of people with Parkinson disease (PwP) to explore their experiences and unmet needs.

**Methods:**

A systematic review for retrieving qualitative studies included six databases: MEDLINE, Embase, CINAHL, Cochrane, PsycInfo, and Scopus. Inclusion criteria were as follows: (i) studies on the experience of caregivers of PwP in the context of STN‐DBS, (ii) English peer‐reviewed articles, and (iii) qualitative or mixed methods studies reporting caregivers' quotations. After the appraisal of included studies, we performed meta‐synthesis of qualitative findings. Descriptive themes and conceptual elements related to PwP partners' experiences and unmet needs were generated.

**Results:**

A total of 1108 articles were screened, and nine articles were included. Three categories were identified: (i) dealing with Parkinson disease (PD) every day (the starting situation characterized by the impact of PD on ordinary life; the limitations to partners' socialization; partners' efforts in stepping aside for love and care activities), (ii) facing life changes with STN‐DBS (the feeling of being unprepared for changes; the fear and concern due to loved ones' behavioral changes; struggling to find an explanation for those changes), and (iii) rebuilding the role of caregiver and partner after STN‐DBS.

**Conclusions:**

This meta‐synthesis elucidates concerns, challenges, and unmet needs of partners of PwP who underwent STN‐DBS. It is important to provide them with information, education, and adequate support to face these challenges. Professionals need to involve partners in the care and decision process, because STN‐DBS‐related outcomes do not depend solely on the well‐being of PwP but also on the well‐being of individuals surrounding them.

## INTRODUCTION

Caregivers and care partners (CGs) are crucial in caring for people with Parkinson disease (PwP) by offering physical, emotional, and economic support and preventing early nursing home placement [1, 2]. Approximately 88% of men and 79% of women with Parkinson disease (PD) identify an informal caregiver [3]. The relationship between caregiving and PD management is complex. PwP CGs must provide a wide range of direct and indirect support in managing the disease. Thus, caring for a person with PD, especially in its advanced stages, can be challenging for the CGs who must devote more time and energy to the loved one. This situation may cause “caregiver burden” (CB) [4, 5], defined as “the level of multifaceted strain perceived by the caregiver from caring for a family member and/or loved one over time” [4].

Subthalamic nucleus deep brain stimulation (STN‐DBS) represents an effective treatment for PwP [6, 7, 8, 9]. STN‐DBS significantly improves quality of life (QoL) in the short and long term after surgery [6, 7, 9, 10]. However, the indirect effects of STN‐DBS on CGs are unclear and understudied [11]. Interestingly, more than half of CGs rated STN‐DBS outcome at 1‐year follow‐up as unfavorable for themselves [12]. In particular, as reported in the quantitative review by van Hienen et al., several caregiver‐related factors may influence CGs' well‐being after surgery, including the preoperative caregiver QoL, the age of PwP, psychiatric rating scales, and preoperative relationship quality scores [12]. Moreover, psychiatric symptoms (i.e., depression, impulsivity, compulsivity, and personality changes) could significantly and negatively influence the post‐STN‐DBS burden [11] expressed by CGs.

Recent systematic reviews highlight the potential risks associated with CGs' dissatisfaction and connubial problems following STN‐DBS. These issues can considerably impact the functioning of the patient‐caregiver dyad and may subsequently affect both parties' QoL. Although STN‐DBS can provide significant benefits in terms of motor symptom management for patients, the psychological and relational aspects of caregiving should not be underestimated [5, 12]. Although these studies outline a general trend of CB after STN‐DBS, they do not look deeply into CGs' experiences and unmet needs, which require a more thorough understanding. For this, we opted for a systematic review of qualitative studies. Qualitative research offers a unique approach to exploring complex and nuanced phenomena, such as individual experiences, social contexts, and cultural influences. By delving into the subjective aspects of the topic, we aimed to uncover more profound insights into the underlying factors that may not be adequately captured through quantitative approaches alone.

In this sense, partners' experiences and perspectives can be studied “in their own words” through qualitative research. Our review question was as follows: “How is the experience of CGs of PwP treated with STN‐DBS?”

## METHODS

Although both systematic reviews aim to summarize existing research, systematic reviews of quantitative studies focus on numeric data and statistical analyses, whereas systematic reviews of qualitative studies delve into non‐numeric data, exploring the richness and complexity of human experiences [13, 14, 15]. This approach is commonly called meta‐synthesis or qualitative evidence synthesis [14]. Our study employed a systematic review to collect all relevant qualitative research involving CGs of individuals with PwP who underwent STN‐DBS. Subsequently, we conducted a meta‐synthesis [16, 17] to amalgamate and interpret the findings. Qualitative meta‐synthesis essentially involves an interpretive integration of qualitative research findings. The methodological indications we followed are outlined by Sandelowski and Barroso [16] and the Cochrane Qualitative and Implementation Methods Group [14], foreseeing a comprehensive and systematic database search, the appraisal of qualitative studies, and findings' interpretative synthesis. We have reported this synthesis according to the Enhancing Transparency in Reporting the Synthesis of Qualitative Research (ENTREQ) guidelines [18].

### Search strategy

For the search strategy, it was agreed to use keywords from three primary domains: PD, intervention‐related (STN‐DBS), and qualitative research keywords. The CG‐related keywords were not used to avoid missing studies in which comments and opinions of CGs could have been reported in patient‐focused studies. Search terms are summarized in Table 1. An information specialist (M.C.B.) performed the literature search in MEDLINE, Embase, CINAHL, Cochrane, PsycInfo, and Scopus. She retrieved research studies published from inception to 23 September 2022.

**TABLE 1:** Search domain and search strategy (MEDLINE/PubMed).^a^

Disease	“Parkinson's Disease” [MeSH] OR Parkinson
AND	
Intervention	“Deep Brain Stimulation” [MeSH] OR "Deep Brain Stimulation" OR "Subthalamic Stimulation" OR DBS OR "Bilateral High Frequency Stimulation"
AND	
Qualitative research design	"Qualitative Research" OR "Grounded Theory" OR "Empirical Research" OR Qualitat* OR Interview* OR Observation* OR "Behavior Observation Techniques" OR Narrat* OR Ethno* OR Phenomenol* OR "Focus Groups" OR “Qualitative Research” [MeSH] OR “Focus Groups” [MeSH] AND “Grounded Theory” [MeSH] OR “Interviews as Topic” [MeSH] OR “Empirical Research” [MeSH] OR “Behavior Observation Techniques” [MeSH]

Abbreviation: MeSH, Medical Subject Headings.

^a^ More analytical database‐specific search strategies are provided in the supplemental material.

### Inclusion criteria and selection

Inclusion criteria for the meta‐synthesis were as follows: (i) studies on the experience of CGs (partners, spouses, family members, significant others) of PwP in the context of STN‐DBS with no other advanced therapies such as intestinal levodopa/carbidopa or apomorphine infusion, (ii) English peer‐reviewed articles, and (iii) qualitative or mixed methods studies reporting CGs' quotations.

Four reviewers (F.C., F.A.M., M.P., C.P.) independently screened titles and abstracts of all studies, then checked full‐text articles based on the selection criteria. The reference lists of the full‐text articles were also searched for additional potentially relevant studies. Any conflict was solved through discussion with seven external reviewers (F.S., M.C.B., V.Fi., L.G., V.Fr., E.M., F.V.).

### Quality appraisal

The methodological quality of the included articles was assessed using the Critical Appraisal Skills Program (CASP), as recently suggested for qualitative evidence synthesis [19, 20]. CASP qualitative checklist tool [19] was initially defined for tutoring novice researchers in evaluating the quality of qualitative studies. Researchers split CASP's ten guiding questions into 30 items to assess the quality of research reporting. F.C. and M.P. independently assessed the quality of the included studies. Any conflict was solved by consulting a third reviewer (F.A.M.). Evidence from moderate/low‐quality studies was incorporated later [13]. See Supporting Information for the CASP analysis of the different articles.

### Data extraction and synthesis

A data extraction table was defined for collecting the following information: first author and date, the country where the study was conducted, aim(s), study design/methodology, sample type and size (number of PwP and CGs, age, and gender), disease or PwP salient characteristics, data collection method(s), data analysis strategy, a summary of findings, and limitations.

For synthesizing the data, we conducted a meta‐synthesis [16] through which we developed a higher‐level interpretation of the findings. This process allows researchers to synthesize qualitative data from diverse sources to generate new insights and explanatory frameworks.

To perform the meta‐synthesis, both first‐order (quotes from the CGs in primary studies) and second‐order constructs [21] (findings descriptions of the authors) were considered. Constructs were manually extracted from each article's “results/findings” sections and inserted into a table by F.C. and M.P. Each quotation/authors' description was independently coded with interpretative labels by F.A.M. and C.P. In addition, they inductively derived subthemes and themes by grouping the labels. Any disagreement was solved through discussion with L.G., F.V., V.Fi., V.Fr., and E.M.; C.P., L.G., F.A.M., and F.C. met to discuss the themes and translate them into interpretative categories. We also determined the frequency of categories among the included studies [22, 23] and the intensity of subcategories (by intensity, we mean the percentage of labels grouped in the subcategory compared to the total number of labels) [23, 24]. Ultimately, an explicative model was generated. In the context of meta‐syntheses, an explicative model is the outcome of a process of interpretation. It serves as a theoretical framework to comprehensively understand the phenomenon under investigation. The final categories and the explicative model are based on the consent of all the authors [16].

### Standard protocol approvals, registrations, and patient consents

This systematic review of qualitative studies was conducted in accordance with the ENTREQ guidelines [18].

## RESULTS

### Literature search and studies' characteristics

**FIGURE 1:**
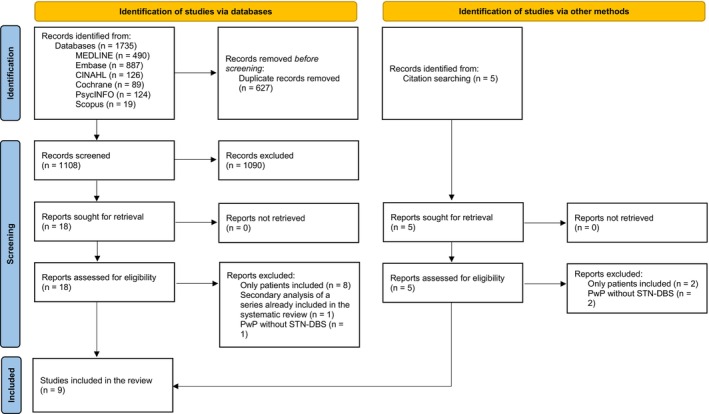
PRISMA (Preferred Reporting Items for Systematic Reviews and Meta‐Analyses) flow diagram (PwP = people with Parkinson's disease; STN‐DBS = subthalamic nucleus deep brain stimulation).

A total of 1735 articles were retrieved. Duplicates (*n* = 627) were removed. Articles (*n* = 1108) were reviewed by title and abstract, and 1090 articles were screened against inclusion criteria and not included. Consequently, 18 full‐text articles were assessed, and five articles were found through the articles' bibliography check. Fourteen records did not meet the inclusion criteria. Nine qualitative studies were finally selected. Figure 1 illustrates the search process through the PRISMA (Preferred Reporting Items for Systematic Reviews and Meta‐Analyses) flow diagram [25].

The nine studies included 117 CGs (68 women, 25 men, 24 unspecified) with a mean age of 59 years (±6.03, median = 60, range = 30–88). In most studies, interviews were performed within the first year after surgery, whereas the follow‐up was very variable (from 1 month to 10 years after surgery). Concerning the critical appraisal (see Table 2), most studies were classified from high/moderate to high quality (*n* = 7/9; 78%), whereas only two were classified as low quality. The main concerns were underexplained methodological choices, ethical issues, and poor discussions of the studies' limitations.

**TABLE 2:** Studies' characteristics and results of the quality appraisal.

First author (date), country	Aims	Study design	Sample type and size (*n*, age, types)	Others clinical characteristics	Data collection method(s)	Data analysis strategy/strategies	Summary of findings	Limitations reported by the authors	Critical appraisal results (score)
Schüpbach (2006) [33], France	To prospectively evaluate the impact of STN‐DBS on social adjustment in PD patients.	A longitudinal prospective study with a mixed approach: qualitative interviews study and quantitative clinical rating scales 18 and 24 months after surgery.	29 advanced PD patients (15 women, mean age = 52.4, ±9.0 years) treated with STN‐DBS. Information about caregivers was N/A.	Advanced levodopa‐responsive PD patients (mean disease duration = 10.8 ± 4.8 years).	Unstructured in‐depth semistructured psychiatric interviews were performed before and 18–24 months after bilateral STN‐DBS exploring work, social life, family life, marital life, and relations with children.	N/A.	Social adjustment did not improve after surgery. Several issues related to social adjustment were observed, affecting the patients' perception of themselves and their body, marital situation, and professional life. Marital conflicts occurred in 17/24 couples. Only 9/16 patients who had a professional activity before the operation went back to work after surgery. After STN‐DBS, patients experienced difficulties in their relations with themselves, their spouses, families, and socioprofessional environment.	N/A	Low (11)
Gisquet (2008) [30], France	To understand personal, familial, and professional difficulties experienced by PD patients with DBS.	Qualitative interviews study, from 1 day to at least 2 years after surgery.	30 patients (13 women and 17 men) between the ages of 39 and 79 years. Only 1 woman as caregiver was included.		Semistructured interviews performed in different time frames: before the surgery, from 1 day to 6 months after surgery, and at least 2 years after the DBS. Interviews explored 4 dimensions: work, social life, family life, and marital relations.	Thematic analysis.	DBS suppresses the most striking symptoms of PD, but at the same time, the patient loses control over managing the illness, over his own life, and experiences significant personality changes.	The eventual opportunity to build a cohort, even restricted, would have allowed questioning the same persons before and several years after the operation.	Low (12)
Haahr (2013) [27], Denmark	To explore the lived experience of being a spouse to a person living with advanced PD before and during the first year after DBS.	Longitudinal qualitative interviews study, collected 3 times during the first year after surgery.	9 spouses of PD patients undergoing DBS. 3 of the spouses were men, and 6 were women. Mean age of the spouses at disease onset = 46 years (range = 27–61). Mean age of the spouses at the time of DBS = 61 years (range = 41–76).		Qualitative in‐depth interviews performed before and 3 times during the first year after DBS.	Thematic analysis applying the hermeneutic phenomenological methodology of van Manen.	The uniting theme "Solidarity–the base for joined responsibility and concern" was the foundation for the relationship between spouses and their partners. Before treatment, the theme "Living in partnership" was dominant. After treatment, 2 dichotomous courses were described: "A sense of freedom embracing life" and "The challenge of changes and constraint."	Small sample size; caregivers were all of the same cultural backgrounds; the first study to apply hermeneutic phenomenological approach to explore the spousal experience of life following their partners' treatment of DBS.	High (27)
Lewis (2015) [34], Germany	To study caregivers' perception of their well‐being 1 year after STN‐DBS in PD patients.	A longitudinal prospective study with the mixed approach: qualitative interviews study and quantitative clinical rating scales performed 3 months and 1 year after surgery.	25 caregivers (18 women and 7 men, mean age = 60.00 [±10.92] years) and their respective STN‐DBS PD partners (18 men and 7 women, mean age = 62.04 [±8.01] years).	The baseline MMSE score of caregivers was 28.96; the baseline BDI‐2 score was 11.54. Of the 25 caregivers, 24 participated at the 3‐month FU and 20 at the 1‐year FU.	Semistructured interviews were performed 3 months and 1 year after STN‐DBS.	Categorization of the semistructured interviews by 2 coders following Mayring's theory‐based content analysis in the negative outcome group or the positive outcome group.	At 3‐month FU, caregivers were more indecisive concerning their well‐being than at 1 year after STN‐DBS. At 1‐year FU, caregivers from the negative group had more significant depression and anxiety, and lower QoL ratings. They were significantly older compared to the positive group. Patients' depression showed significantly more substantial improvement in the positive outcome group. At 1‐year FU, >50% of the caregivers rated their subjective well‐being as negative. Especially older and more depressed caregivers are at risk.	Small sample size.	Moderate to high (27)
Liddle (2018) [29], Australia	To explore the occupational experiences of PD patients undergoing STN‐DBS.	Structured qualitative description study, time since DBS = 2–60 months.	14 PD patients (4 women, mean age = 64, range = 47–75 years); 10 family members of patients (6 women, mean age = 65, range = 43–72 years); 11 clinicians.		Semistructured interviews.	2 members of the research team read the transcripts. All transcripts were entered into Dedoose and were independently coded. Transcripts of different stakeholder groups were not separated for analysis, as they reflected complementary areas of core experience during initial coding. 3 broad themes emerged from analysis; 2 of the themes reflected transitions experienced through occupation, and 1 reflected the changing interpersonal aspects of occupation. Peer checking occurred throughout analysis and coding through the discussion of emerging thematic areas. Discrepancies were discussed resulting in adaptation and simplification of the coding tree into 2 themes.	Occupations emerged as a key aspect throughout the DBS experience. Occupation seemed to act as a self‐generated barometer for the experienced changes and a means of understanding both disease processes and treatments. Occupational experiences and performances shaped people's understanding of their condition, the impact of treatments, and their overall adjustment; and shifting occupational identity, where the life transition of DBS altered the occupational experiences of relationships, volition, roles, and responsibilities of people with PD and their family members.	Generalizability of the findings; selection bias (people who could not communicate easily in English were excluded, thereby not capturing people's perspectives from different language and cultural backgrounds); retrospective recall.	Moderate to high (26)
Liddle (2019) [31], Australia	To explore the adjustment and associated education and support needs of people with PD undergoing DBS and their family members.	Structured qualitative description study. Interviews performed from 1 to 36 months after surgery.	14 PD patients (4 women, mean age = 66, range 47–75 years); 10 family members of patients (6 women, mean age = 65, range = 43–72 years); 11 clinicians.	Advanced levodopa‐responsive PD (mean disease duration = 8, range = 4–14 years).	Semistructured interviews. Caregivers with PD and family members were asked about their lived experiences, including the decisions to make, and experience of DBS, which types of supports and strategies they used or would have liked, and their past or ongoing support needs.	The inductive analysis involved transcripts being read for familiarity and summarized individually to understand each participant's experiences and develop preliminary content categories.	Needs for peer‐based education and realistic, meaningful goal setting; clinical support related to the surgery and support for the person and their family around immediate changes experienced; timely clinical and practical support for the person and family around new changes and challenges to symptoms, behaviors, and roles; direction and support for reengagement in the self‐management of the condition, and current and future changes related to the disease. All caregivers with PD and their family members in this study indicated that their experiences with DBS had led to positive changes in their symptoms and lives.	Selection bias (people who could not communicate easily in English were excluded, thereby not capturing people's perspectives from different language and cultural backgrounds); retrospective recall.	High (26)
Mosley (2019) [32], Australia	To explore the meaning and significance of stimulation‐related neuropsychiatric symptoms among a sample of PD patients and their spousal caregivers.	Qualitative interviews study; interviews were conducted 6–12 months postoperatively.	10 persons with PD treated with STN‐DBS (9 men, 1 woman, mean age = 59.4, range = 36–71 years) and their 10 spousal caregivers (9 women, 1 man, mean age = 57.9, range = 35–70 years).	PD patients diagnosed according to the UK Queens Square Brain Bank criteria treated with bilateral STN‐DBS who developed psychiatric symptoms due to stimulation.	Semistructured qualitative interviews exploring psychiatric symptoms attributable to STN‐DBS and its impact on autonomy, identity, and responsibility.	Thematic analysis.	Neuropsychiatric symptoms highly burdened caregivers; both patients and caregivers felt unprepared for their consequences, despite receiving information before DBS, desiring greater family and peer engagement before neurosurgery. Caregivers held conflicting opinions as to whether emergent symptoms were attributable to neurostimulation. Many felt that they reflected aspects of the person's “real” or “younger” personality. Those caregivers who perceived a close relationship between stimulation changes and changes in the mental state were more likely to view these symptoms as inauthentic and uncontrollable.	Bias toward men; most patients (90%) were men. Possible selection bias in the definition of “STN‐DBS induced” psychiatric symptoms.	High (29)
Thomson (2020) [26], Australia	To examine the significance and meaning of DBS‐related changes in personality and self for patients and caregivers.	Prospective qualitative study, 9 months follow‐up after surgery.	22 caregivers (11 patient–caregiver dyads). Patients: 7 men and 4 women, 45–73 years of age. Caregivers: 2 men and 9 women, 51–69 years of age.	PD patients: 6 were employed, and 5 had retired. Time since PD diagnosis ranged from 3 to 12 years. Caregivers: 9 spouses, 1 parent, 1 child.	In‐depth, semistructured interviews performed before and 9 months after DBS.	Thematic analysis.	3 themes present before DBS were identified, which reflected a time of anticipation, whereas 3 themes present after DBS reflected a process of adjustment. Post‐DBS changes in personality were experienced. Personality changes directly affected caregivers and were most pronounced when associated with disease progression. The negative influence of illness on patients' personalities and sense of self was apparent. For some, DBS facilitated the restoration of the patient's premorbid self. Perceptions of control were also relevant to patients. Patient and caregiver awareness of personality change as a post‐DBS risk appeared limited.	All caregivers were inhabitants of the same region, and only patients undergoing DBS through the private health care system were included, which affects generalizability.	High (29)
Chacón Gámez (2021) [28], Switzerland	Collecting and analyzing a wide range of experiences of PD patients treated with DBS and their family caregivers.	Multimodal approach, qualitative study by means of narrative semistructured interviews and drawings collected from 1–10 years after surgery.	19 patients with PD and DBS (6 women, 13 men, mean age = 67.2 years, range = 54‐75) and 17 family caregivers (13 women, 4 men, mean age = 64.2, range = 30–88 years).	Average years with DBS for patients = 1–10, mean = 4.7.	Narrative semistructured interviews and drawings.	Hybrid process of inductive and deductive thematic analysis.	7 principal themes have been identified: “Everyone's Parkinson's is different,” “Changing as a person during the disease,” “Going through Parkinson's together,” “DBS improved my life,” “I am treated with DBS but I have Parkinson's still,” “DBS is not perfect,” and “Being different after DBS.” PD is perceived as an unpredictable and heterogeneous disease that changes from person to person, as does the effect of DBS. Although DBS side‐effects may have an impact on patients' personality, behavior, and self‐perception, PD symptoms and drug side effects also have a great impact on these aspects.	The study did not focus specifically on 1 issue in relation to PD or DBS as other studies did. Therefore, some issues (i.e., caregiver burden) may have been missed during the interviews. The caregivers were interviewed only after being treated with DBS and not before (recall bias). Other issues need further elucidation, such as patients' and caregivers' experiences with the side effects of dopaminergic treatment or the impact of memories of DBS surgery.	High (28)

Abbreviations: BDI‐2, Beck Depression Inventory II; FU, follow‐up; MMSE, Mini‐Mental State Examination; N/A, not available; PD, Parkinson disease; QoL, quality of life; STN‐DBS, subthalamic nucleus deep brain stimulation.

### Meta‐synthesis findings

The experience of CGs was categorized into three categories: (i) dealing with PD every day (the starting situation characterized by the impacts of PD on ordinary life [intensity: 9.6%; labels: *n* = 10/104], the limitations to CGs' autonomy and socialization [intensity: 12.5%; labels: *n* = 13/104], and the CGs' efforts in stepping aside for love and care activities [intensity: 12.5%; labels: *n* = 13/104]), which referred to the PwP's conditions before surgery; (ii) facing life changes with STN‐DBS (the feeling of being unprepared for changes [intensity: 9.6%; labels: *n* = 10/104], the fear, and concern due to partners' behavioral changes [intensity: 11.5%; labels: *n* = 12/104], and struggling to find a rational explanation for those changes [intensity: 5.8%; labels: *n* = 6/104]); and (iii) rebuilding the role of CG/partner after STN‐DBS. This last category includes learning how to cope with changes (intensity: 14.4%; labels: *n* = 15/104) and two possible relational outcomes: renaissance (i.e., the rediscovery of partners' own life in a new couple's relationship [intensity: 16.4%; labels: *n* = 17/104]) or estrangement (when loved ones had behavioral problems and/or showed PD exacerbation, which added to CGs' disappointed expectations and hopes to return to as before [intensity: 7.7%; labels: *n* = 8/104]). The meaningful quotations are reported in Table 3, and a detailed description of categories, subcategories, and participants' quotations is reported in File S4.

**TABLE 3:** Meaningful quotations.

Categories	Subcategories	Narratives
Dealing with PD every day	Impacts	“We have some great‐grandchildren, and he loves them. They know when he starts making faces and starts going OFF, then his face ‘dies’ too and they leave him alone.” [27]
		“He'd stay at home most days and watch TV all day. That was starting to bother me. Because that's not who he is.” [29]
		“This has changed a lot, the disease. That is quite clear. Then, as you can see with the disease, where dyskinesia became more and more pronounced, comes the physiognomy change. I no longer knew my own wife by her face. It was so bad, the disfigurement that was caused by this illness that affects not only the movements but also the face.” [28]
		“Parkinson's is like a cage for the person. The person is like in a cage for me and I cannot get into this cage.” [28]
		“He was so hyperactive, and I did not know if that was because he knew that he had a disease and he wanted to enjoy life (…) He was thinking more about himself, looking more for his own pleasure. He had no sense of time, and he was looking for his pleasure. That was his first concern, to think of himself.” [28]
	Limiting autonomy and socialization	“Caroline stated: ‘We went to the party, but when we had finished dinner, he got cramps in his legs and went out and sat in the car. Nothing worked, and we went home. It is frustrating. You miss the rest of the party… dancing and such. I would not dream of sending him home in a cab and stay at the party.’” [27]
		“Mary reflected: ‘There are so many things he would like to do and knowing he wants to so much, I cannot bring myself to go, I cannot do that.’” [27]
		“Christine said: ‘There has been loss of many things. Loss of the person you married, loss of abilities, loss of strength…I have experienced social isolation. Having to find a new identity, a new social life, a new way of living life.’ She continued: ‘We have talked about what possibilities we have. How can we compensate for what we have lost?’” [27]
		“I think the disease itself is the devil on one side and the beautiful fairy on the other side. It is entirely day‐dependent, time‐dependent. Sometimes you could almost despair about the disease and other times, everything is quite normal and you can say to yourself that everyday life is actually quite normal. And then in the next half hour nothing works at all. What is also the problem is that we have to prepare every appointment very carefully.” [28]
		“For example, when we were at the table with the children, he ate a lot and very quickly, and then stood up and went to his computer. And that's difficult with children, when you try to educate the children and say: wait until everyone has finished and then you can get up and leave the table. And he, he had no concept of parenting anymore.” [28]
	Stepping aside for love	“Mary said: ‘We have known each other for so many years and still love each other very much. And THAT'S the crux of the matter that we care so much for each other that we are positively in this together!’” [27]
		“Victoria described a need to be strong: ‘I often feel I have to be the strong one. And often, he says to our friends that I am so strong. But deep down I am not. Sometimes I have the need to talk to somebody, as well.’” [27]
		“I feel sometimes pressured because I make an appointment for her somewhere and calculate how long we need to get her ready and to be there. And then when we leave, it can happen that nothing works until we get to the station because it takes us a quarter of an hour to walk ten meters and then the train bye.” [28]
		“And yes, how should I put it, um, because of the illness she has also become more selfish. So, she comes first and then again and then maybe the others. Sometimes I've also said, I'm not a domiciliary care provider. You pay him and you can give him orders, but I do not get paid.” [28]
Facing life changes with STN‐DBS	Feeling unprepared	“Had they said to me he may have a change of personality, then I could have said well this has happened, and got on to it sooner. From my point of view, I've had to learn the hard way about the side effects.” [32]
		“We have not done any counselling at all, and I think we need to. As a spouse, you need to be prepared that these things can happen, and that husbands or partners can turn feral [wild] and not to—we were told not to take it to heart—whatever is said is said out of—they cannot help it. But in saying that, that's kind of not enough. You still hold—I mean, I do—I still hold on to things that were said and things that were done because it's ultimately affected our relationship. That's something that I have to move on from but it's really difficult.” [32]
		“Definitely the behaviour side of it… because that was really quite scary… he would just go! Whatever he'd thought he'd just go and do it… We had no understanding that could just be changed by changing the controls… So, I think they need to tell people that, because if it had gone on and just let him do whatever… Well, I wondered why—every time you go to a neurologist appointment, they'd ask you, ‘Oh, is there any change in behaviour?’… gambling, sort of, alcohol‐type behavior… I'm going ‘No’… then when that happened… ‘Oh, now I know why you ask that all the time!’” [27]
		“I think both people need to be well informed. I think that's important and even other family members and or friends. I really went out of my way. Dianne does not even know this, but people would send me stuff about Parkinson's, and I'd likewise send it out to other people so that everyone had a bit of an understanding.” [31]
		“Does the medicine work right away? Does not it work yet? Uh, a lot of things are happening at the same time. It's very difficult and each person is very different.” [28]
	Experiencing patients' behavioral changes with concern and fear	“Hmm, hmm. Yes, well, he gets us into trouble, or he could get us into trouble, with things that might cause us problems.” [34]
		“He is not like he was before. He gets annoyed over the smallest things. He is almost aggressive towards me sometimes. I do not understand.” [30]
		“Seriously hyper, that really concerned me.” [31]
		“Family members were more likely to describe some concerns about the way in which the changed symptoms related to changes in daily life. Some expressed concern that the person with PD was not doing more and embracing independence, while others were concerned about the magnitude and rapidity of the changes experienced.” [31]
		“I'd wake up, and I'd hear this noise, and he'd be scrubbing the skirting boards.” [29]
		“He talked incessantly, non‐stop, and just kept swapping from topic to topic to topic… He'd just ring people up and go, ‘Oh, I'll pick you up in 10 minutes’… but the neurologist just changed channels or whatever, and that disappeared… Apparently, he was on a high with it. It was like being on drugs and stuff.” [27]
		“My biggest fear… I can cope with absolutely anything. If he's quadriplegic, it's fine, I can deal with that, but I cannot deal with—the psychiatric changes, it scares me too much. How he behaved, how he was when he was back there, I cannot do that again.” [32]
		“He really had a personality change for a short period of time and also a maniac phase. He was completely different for a while.” What do you mean by manic phase? “Yes, after the operation he was really changed in his manner, that he for example/that he complimented me or hugged me when greeting me, as he never did before (…) He bought an expensive watch and booked holidays, big holidays, without discussing it with my mother. And also wanted to write a book. Yes, things like that.” [28]
	Struggling to find an explanation	“Susan said: ‘We have been told that his speech can be affected, but you get frustrated anyway and find it hard to understand… because I am not ill.’” [27]
		“I've seen with this that people can change pretty quick just from a wire. Same person, same mind, or same brain, just shift a bit of voltage somewhere and a different person … But I do not see why I should condone bad behaviour. Whether you are crook [ill] or not, bad behaviour is bad behaviour.” [32]
		“She was always a bit feisty beforehand, but now… She does get very defensive very quickly… It could be a mixture I think, of the DBS and muddling with her brain, and the fact that her Parkinson's has progressed, and the [cancer‐related] operation. Whether [the cognitive changes] were just marred by the movement prior to and you concentrate just on one thing and forget about the others…because we concentrated so much on the movement and trying to help with that.” [27]
Rebuilding the role of CG/partner after STN‐DBS	Learning to deal with changes	“There's going to be more stressful times, I'm sure.” [31]
		“This man, this personality changes he's gone through, it's crazy. It's not—he's not the man I married. He's definitely not the man I married. He's changed so much. If that's just part and parcel of Parkinson's, I guess?” [27]
		“Caregivers spoke of their partner ‘no longer being the person I married.’” [32]
		“We call him the Energizer Bunny, and when the friends walk in, they'll say to him, are we switched on today, or switched up, because he's just got this energy. Then when you turn him down… in the afternoon, he'd have to have a little nap. Well, he does not like that. He likes to have this Energizer Bunny energy. Since he's had a taste of it, he really likes it. It's almost like an addiction, actually… to me, it's almost like control.” [32]
		“Ever since the operation, I feel lost. Before, when he was sick, we were a perfect couple. Now, he wants to live the life of a young man, go out, meet new people, all of that is intolerable! I would rather he be like he was before, always nice and docile!” [33]
		“I was used to doing a lot of the jobs myself [before surgery], then he'd come in, and he'd start doing things without any sort of consultation. The first six months were pretty rocky.” [31]
		“Any work colleagues or friends of his [say], ‘It's great to see the old [patient name] back, we were really worried there for a little while’… now I feel like I'm not totally, you know, he's back, so I'm not alone again. So that's good.” [27]
		“It's better, but you still live next to a sick person, and you sleep next to a sick person.” [28]
	Renaissance	“I feel like I have got a new husband… we are much closer… it is almost like being in love again.” [27]
		“Altogether we are happy with the treatment. I am probably the one who is most happy. It is a paradox, but I think so. In some ways I have got my husband back.” [27]
		“When I saw him, it was just like it was almost a miracle because he had the typical frozen face. And I looked at him in the chair, and his face was alive again. Unbelievable.” [31]
		“If he did not have the DBS, then he would have been excluded from a lot of it.” [29]
	
		“After DBS Mary said: ‘…we do not feel disabled as we did before DBS.’” [27]
		“Christine said: ‘I am very alert that he does not get too comfortable… such as he asks me to get him things and I tell him to get up and get them himself,’ and Helen said: ‘Now he has to take care of his medicine himself. I used to do that, but I do not anymore… I keep an eye on him, but he does not know that.’” [27]
		“DBS ‘gave me a peace of mind for me that he can dress himself, shower himself and cook for himself.’” [31]
		“Since then, she can use her hand completely again. She does not tremble. She can do different things by herself again. Before I had to cut the meat and everything for her, and today everything is back to normal.” [28]
	“No, nothing bothers me about her, even that she has such a device above her chest that you can see and feel, that does not bother me. (…) That belongs to my wife. Exactly. It's not a foreign body from my point of view. I do not perceive her as my wife, who has electronics in her brain. I just do not think about it at all.” [28]
	Estrangement	“Susan reflected on: ‘Suddenly there is nothing there for us to look forward to. We have to deal with the situation as it is.’” [27]
		“Susan said: ‘I am really tired. I really am. A lot of things have happened, and as they [doctors] tell us, the illness is still progressing.’” [27]
		“Not really. He thinks it's his business (referring to gambling via telephone) and that he has the responsibility, and that I have nothing to do with it. I tell him that I do have something to do with it, because if he does something like that and they clear out our bank account… then I've got as much of a problem as he has.” [34]
		“C. How should I say this… he is very lazy. No energy at all, nothing. […]. I go to work in the mornings, and when I get home, I have to do all the housework. He does no hoovering, no dusting, nothing at all. Do you see? He gets up after me in the mornings, but he does not open the blinds or lay the table. Nothing, even though these are only small chores.” And when you ask him about this, is he at all reasonable about it? “C. Well, yes, he says, ‘I'll have to change something.’ But nothing happens, and he does not change anything. My view is that, strictly speaking, if I did not swallow everything, then we would get into a fight every day—and that would not be a life worth living anymore.” [34]
“It is noticeable today that everything has become a little slower (…) The asking back and forth, that has increased. In the past she cooked, I had no problem, I ate what she made. Today I have to ask her, what would you like for dinner today? That has become our daily routine, three times a day, or, in the morning, I say, what would you like, bread, everything, at noon and in the evening. Yes, that has become my task, to think a bit more for my wife as well.” More after the intervention than before? “Yes, before I did not have to think for my wife anything. She organized everything herself and was independent in every way. She managed the household, but today we have to share everything.” [28]	

Abbreviations: CG, caregiver; PD, Parkinson disease; STN‐DBS, subthalamic nucleus deep brain stimulation.

#### Dealing with PD every day

This category summarizes the experiences lived by CGs during the phases that preceded the STN‐DBS and were mainly associated with the daily problems related to the disease.

##### Impacts

In three studies [26, 27, 28], CGs described their experience of living with PD before STN‐DBS. CGs revealed events and situations of concern and discomfort in which they began to realize that something was wrong [28], to the point of no longer recognizing their partner. For example, dyskinesia impacted the physiognomy of the loved ones, who could not control movements and were “glassy‐eyed” [28], triggering fear and concerns. Family relationships changed between partners and children/grandchildren [27] because, as described [28], loved ones' empathy and self‐awareness diminished with losing their sense of reality over time.

After receiving the diagnosis, some CGs verbalized PD as “autumn […],” “the end of a life”[…] “time to mourn,” and “a cage for the person” that CGs could not get into [28].

##### Limiting autonomy and socialization

In addition, CGs' narratives reported fatigue and strong social constraints for them [28] due to the limitations of the disease, which limited the autonomy and socialization of CGs. Going out as a couple was considered very important, but it required much planning and could cause frustration. Reported unmet needs concerned the possibility of having space for oneself, self‐care, and communicating one's emotions or state of mind.

##### Stepping aside for love

Concurrently, some CGs reported a selfish turn of their loved ones, whereas others described that they were rarely sad in front of their partner, trying to step aside for love. Over time, partners became CGs and loved ones restless and discontented PwP.

#### Facing life changes with STN‐DBS

Under this category, narratives concerning how CGs signified the treatment‐related changes were collected. Three subcategories, inextricably intertwined with each other and shaping the CGs' perspective, were defined: feeling unprepared, experiencing PwP's behavioral changes with concern and fear, and struggling to find an explanation.

##### Feeling unprepared

CGs reported that they generally felt unprepared [29, 30, 31] for what STN‐DBS would entail in terms of both the amount of information they received and the side effects. Some CGs stated they had to recognize the STN‐DBS effects at their own expense by experiencing them. One spouse described feeling in a “trial and error” situation every time neurologists had to adjust STN‐DBS parameters [32]. A participant perceived the patient as under the influence of drugs [26] before STN stimulation programming optimization. This feeling shaped the preoccupation and fear of not managing their loved one's behavioral changes.

##### Experiencing PwP's behavioral changes with concern and fear

A cross‐cutting aspect of the CGs' narratives related precisely to the rapidity with which PwP demonstrated a sudden escalation in mood (aggressiveness described by one participant as the “turning feral” of her husband) [32]. The concern was related to when the changes were acute and rapid. The unpredictability of these behavioral changes exacerbated some CGs' feelings of helplessness and frustration [28]. Many even recounted being afraid they could not handle the personality changes, unlike the motor changes they were accustomed to.

##### Struggling to find an explanation

Transversely, across the data, CGs had difficulties accepting changes in their loved ones [26, 27, 32]. At the same time, CGs reported struggling to explain the reasons for these changes. Narratives fluctuated between blaming the treatment or charging destructive behaviors to the PwP's will. The behaviors were seen as artificial, like the effect of a drug, or as internally motivated. In one case, a CG explained the behavioral consequence of STN‐DBS as an amplification of her loved one's past conduct.

Interestingly, these CGs' feelings and experiences did not appear to be associated with the success of the surgery regarding motor symptoms. Although CGs noted improvements in PwP's symptoms and quality of life post‐STN‐DBS, they still faced ongoing challenges. These included postoperative neuropsychiatric symptoms, mood swings, marital conflicts, and a lack of clarity about STN‐DBS' effects.

#### Rebuilding the role of caregiver and partner after STN‐DBS

This category summarizes the experiences linked to the caregiver's need to reconstruct his/her role as both caregiver and partner following STN‐DBS changes, which have significantly affected the PwP's clinical conditions and the degree of autonomy.

##### Learning dealing with changes

The previous category introduced a critical aspect that is now emphasized in this last category: the problematic acceptance of the changes brought by STN‐DBS in the relationship with the loved one. The CGs participating in the studies needed to reshape the relationship's dynamics and roles, as they were inevitably confronted with various changes.

CGs struggled to adjust to a “new partner” after STN‐DBS [26, 28, 32] like the ones who wanted to have “the life of a young man” [33], while also revealing fear about increased stress due to the change [31]. CGs' perceptions shifted from the very early to the maintenance post‐STN‐DBS period.

Right after the post‐STN‐DBS period, while patients were adjusting to reconnecting with their new bodily sensations [34], CGs tried to push them into doing new things (or things they were used to doing before STN‐DBS). In this period, some stated that they no longer recognized their partner and were afraid of their erratic behavior [26, 32], even doubting whether they had done the right thing in undergoing STN‐DBS treatment. The “new energy” acquired after surgery was not well seen by some CGs [32], who had to explain the strange behaviors of their loved ones to their children. Some partners felt destabilized by the results of the STN‐DBS and were nostalgic for the couple they were [33]; they felt thrown around in multiple different roles in a short period [32]. In this sense, many CGs described the lack of control they were used to having, complaining that PwP were doing things without consulting the partner anymore [29, 31, 32]. Within the 6 months after the surgery, many of the included articles' results report a general worsening of relationships within couples [27, 30, 33, 34] except for children who felt relieved for the regained autonomy of their parents [29, 31]. Over half of the CGs participating in the Lewis et al. study rated the STN‐DBS outcome at the 1‐year follow‐up as unfavorable for themselves [34]. At the time of the first follow‐up, the CGs were preoccupied with the patients' assistance and gave less consideration to their well‐being. This period was crucial for CGs and PwP, and what discriminated the relationships was experiencing a new partnership and sharing or not. To cope with this new condition, CGs in the included studies could find two solutions; in the first, CGs managed to reconfigure their role in a way they considered positive, gaining autonomy and, at the same time, a satisfying couple life; in the second, CGs tried to put up with their loved one, resigning themselves to not having personal independence and to having lost, in addition to their role, also their partner.

##### Renaissance

With CGs finally free to reconfigure their role and start to look after themselves, PwP's functioning status also positively impacted CGs' perceptions as STN‐DBS' benefits reduced the stress of organizing home care [33], allowing them to restart traveling [27] and reducing the sense of burden [26]. Some CGs said they were reborn as a couple, in mutual love, and in finding one's partner again. It was reported that STN‐DBS was miraculous [31] in fixing physical/motor aspects [27, 31] and restoring complicity in the relationship [29]. One study reported that CGs were important in persuading PwP to participate in social life [27]. Partners felt not “disabled” anymore as a couple [27].

Similarly, CGs reported that they regained their role, freedom of movement (forced scheduling of outings, for example, was lacking), and occupations (including work activities, leisure, and social gatherings) [27, 29, 31, 32, 33]. CGs reported that their loved ones were more independent in dressing, cooking, and managing medications [27, 31]. In this direction, CGs reported feeling good partnership and solidarity, being able to do things together, and being back as a team [26].

##### Estrangement

Conversely, CGs of patients with cognitive issues, prior psychiatric history [32], and poor STN‐DBS outcomes experienced an increased burden [28]. The included articles have reported that after 12/24 months after surgery [27, 33], many couples, regardless of preexisting marital issues, entered a crisis. Around the follow‐up, partners became depressed, divorced, or rejected by their spouses [33]. Adjustments to STN‐DBS‐related changes did not occur for CGs whose loved ones were experiencing an exacerbation of PD [27], causing a sense of estrangement or a worsening of prior/preexisting psychiatric symptoms [32]. Moreover, CGs reported their disappointment for dashed expectations: some expected their partners to have a renewed vitality [28, 33, 34]. Some CGs describe how they no longer have patience with the unruly behaviors that, despite the treatment their loved ones had received, they continued to exhibit [34]. Without the perception of being a team, doing things together, and making shared decisions, one caregiver said, “Why should I condone bad behavior?” [32].

## DISCUSSION

This qualitative systematic review, focusing solely on CGs of STN‐DBS‐treated PwP, provides insight into their experiences throughout the DBS journey. STN‐DBS is a complex intervention with unexpected postoperative challenges extending beyond clinical outcomes. It affects the PwP and their partner, transforming the roles of CGs and the dynamics of the couple's relationship. It extends its impact to responsibilities within the family, at work, and in their roles as parents. CGs need to change and adapt the way to perform family duties, leisure, and social activities [35, 36, 37].

Before STN‐DBS, social restrictions for CGs relate to PwP's difficulties in eating and motor fluctuations (the couple had to program social activities according to the timeline of motor fluctuations during the day), and social embarrassment over visible PD symptoms [37, 38], which may also shape social stigma [39]. Social isolation could lead to frustration and tension within the couple [40, 41]. Nonmotor symptoms (especially apathy, present in 17%–70% of PwP) [42, 43] are initial components of CB, aligning with a recent meta‐analysis highlighting how nonmotor symptoms consistently contribute more significantly to CB than motor symptoms [44].

After the treatment, although its primary aim often revolves around addressing motor symptoms, it is vital to acknowledge that CGs report enduring persistent challenges contributing to CB [5]. These challenges include the emergence of postoperative neuropsychiatric symptoms and mood fluctuations in their loved ones, marital conflicts, an incomplete understanding of the symptomatic nature of DBS therapy, and limitations on social activities due to these conflicts [11, 12]. Paradoxically, interventions like STN‐DBS, intended to ease the family's burden, can sometimes strain relationships further.

In this regard, our data report that a relational readjustment, whether positive or negative, occurred after surgery, with the possibility that, by developing conjugal conflicts hard to solve, CGs might leave or be left by the partner [45].

Our results show that when the relational readjustment is conflictual, partners need to redefine their role quickly, as noted elsewhere [45]. The solidarity and responsibility between the couple, which were fundamental during the relationship before surgery, according to many of the primary study participants, may no longer be needed by the patient who, thanks to the clinical improvement brought about by STN‐DBS, may become independent from the partner. Nonetheless, PwP need the assistance of partners as DBS postoperative traditional clinic management contemplates repeated visits for DBS programming [46].

In this journey, CGs believed they had not been adequately informed about the possible changes after STN‐DBS. As suggested [47], more attention to delivering education and information about the treatment‐related changes should be paid, keeping in mind that approximately 10% of persons treated with STN‐DBS can develop unintended mood and behavioral changes [48] (e.g., euphoria, irritability, pathological gambling, hypersexuality, impulsivity, but also apathy). CGs of PwP with these symptoms experienced a more substantial burden [11]. STN‐DBS can increase apathy and may cause CGs' distress, as it correlates with higher levels of CB [45]. As mentioned, partners struggled to find an etiological explanation for those neuropsychological changes that were transient [10].

As per a survey study by Hermanowicz and colleagues [49], it was found that nonmotor symptoms had a more significant impact on QoL for 58% of CGs compared to motor symptoms. Additionally, they emphasized the need for more information about these symptoms and their management, underscoring the importance of caregiver education to provide this essential knowledge. Partners should be informed [44] and, if needed, have psychological support.

In the context of PD, programs have been validated to support patients and CGs [50, 51, 52], offering valuable resources that can serve as references for those dealing with STN‐DBS. However, it is worth noting that none of these programs is specifically tailored to the unique challenges posed by DBS treatment. A specific educational program has been developed by surgical teams in France (ParkEduStim), which might help to align patient expectations with potential results from surgery [47, 53]. There remains a clear need for developing and implementing future programs specifically designed to address the unique challenges CGs face in the context of DBS treatment, informed by CB‐related evidence.

In this regard, it is worth noting that most partners in the surveyed literature were women, highlighting the need for a gender‐specific approach to addressing CB, considering the gender disparities in PD prevalence, treatment access, and caregiving resources. The CGs of the primary studies were mostly women. PD is more prevalent in men aged 60–79 years [54]. Nonetheless, when approaching CGs' gender differences, gender disparities in managing and treating advanced PD, particularly regarding the access to DBS [55] and the caregiving resources (lower for women than men with PD) [56, 57] should also be considered. This emphasizes the need for a novel approach to address the CB with a gender‐specific focus.

### Strengths, limitations, and research future directions

This is the first systematic review of qualitative literature focusing on the sole perspective of CGs of PwP treated with STN‐DBS. Other available PD‐related reviews have neglected the voice of CGs in their uniqueness [58] or specifically STN‐DBS [59, 60].

Some limitations should be noted. We focused the search only on peer‐reviewed studies within language and publication limits, possibly increasing the risk of publication bias. Qualitative reseach on this topic is scarce, so we did not exclude studies with low‐quality assessments to maintain the study sample size (117 CGs) and valuable insights as much as possible. The authors screened all possible interpretations during analysis, reaching an agreement. Nonetheless, we did not cover all the possible ways to interpret partners' voices. Furthermore, the follow‐up variability after surgery was large, thus representing another possible limitation of the review's findings. We may assume that the perception of DBS burden and benefit may change over time from the first months after surgery, a more stressful time, compared to the “steady‐state” after programming optimization.

Future studies are needed to support or confirm these findings through proper, high‐quality qualitative and quantitative studies with optimal follow‐up throughout the DBS journey for PwP and CGs.

The included studies in our review do not have generalizable samples in terms of cultural belonging, ethnicity, socioeconomic status, or social determinants. This limitation is primarily linked to the inherent characteristics of qualitative studies. Qualitative research aims to explore the depth and nuances of individual experiences. Therefore, although the findings provide valuable insights into the participants' experiences, they may not be broadly generalizable to larger populations. Moreover, disparities in healthcare access, frequently tied to socioeconomic factors and the accessibility of specialized care (namely, the availability of DBS as a treatment choice), inherently introduce selection bias within the included studies.

However, a prevalent issue persists within the scientific literature focused on PD across both quantitative [61] and qualitative studies. This pertains to the limited attention given to research samples' demographic, social, and cultural diversity. Quantitative and qualitative studies have often failed to prioritize including participants from diverse backgrounds adequately [61, 62]. It is imperative for future research endeavors to proactively address this gap by intentionally incorporating a more diverse range of individuals in the study samples.

In addition, research on PD has been primarily conducted in North America and Europe [62]. This overlooks individuals without access to medical care or treatments, especially in low‐to‐middle‐income countries. Moreover, cultural treatment practices and varying healthcare access worldwide have been largely unexamined. Therefore, there is a crucial need to investigate PD caregiving in diverse regions, as PD rates are expected to rise due to an aging population. Including individuals with PD from different cultural backgrounds in research can lead to early identification and tailored interventions for CB. Understanding the experiences of PwP partners can inform evidence‐based support. Cross‐cultural comparisons may offer insights into alleviating psychosocial CBs [45].

### Clinical implications

Regardless of its success, the potential increase in separations post‐DBS underscores the need for greater psychosocial support in the PD journey. Counseling should be tailored to anticipate challenges partners face before [47] and after surgery [50].

Acknowledging the gender imbalance among CGs, with a majority being women, health care providers (HPs) should adopt gender‐specific approaches to address CB, offering customized support and resources to meet the unique needs of male and female CGs. Clinical practice should involve a comprehensive assessment of CB [4], considering both motor and nonmotor symptoms, social restrictions, and potential relationship conflicts.

Educational programs play a crucial role in aligning patient expectations with potential surgical outcomes, ensuring that both PwP and their partners have realistic expectations regarding the impact of STN‐DBS on both motor and nonmotor symptoms. Moreover, acknowledging the possibility of postsurgery relational adjustments and conflicts within couples, HPs should consider providing long‐term follow‐up and support to assist PwP and their partners in successfully navigating these changes. In conditions like PD, efforts to address CB often focus on supporting them rather than helping them understand how the disease has changed them and will continue to change them, potentially in ways they may not consciously desire.

In light of these considerations, it has been suggested that the existing postoperative care model for DBS may need to be revised [46]. Frequent clinic visits can present challenges, particularly for those living far from a DBS center and experiencing relational difficulties. Recent evidence demonstrates that home‐based interventions can deliver effective care, offering a more accessible and supportive approach to addressing these unique challenges [46, 63]. Additionally, CGs can perceive home care as providing supplemental psychological support by HPs. This personalized approach in the comfort of one's home can enhance the overall caregiving experience and offer valuable emotional and practical assistance [63].

Incorporating these clinical applications into the care of PwP undergoing STN‐DBS can enhance the overall treatment experience, improve partners' well‐being, and contribute to better patient outcomes.

## CONCLUSIONS

This systematic review of qualitative literature, focusing exclusively on the perspectives of CGs of PwP treated with STN‐DBS, provides valuable insights into the caregiving journey. Three main domains emerged, representing the phases before and after surgery. Before surgery, CGs faced challenges related to the disease's impact on daily life, limitations in socialization and autonomy, and their dedication to caregiving. After surgery, CGs grappled with unexpected changes in their loved ones' behavior, which led to fear and concern. They also had to adapt and reconfigure their roles as CGs. Importantly, these domains are interconnected from the CGs' viewpoint.

STN‐DBS is a complex intervention; partners felt inadequately informed about potential changes after the surgery. Given the possible emergence of mood and behavioral changes in PwP following DBS, improved education and information‐sharing regarding treatment‐related changes are crucial. Psychological support and specific educational programs can be beneficial in aligning CGs' expectations with surgical outcomes.

Moreover, CB extends beyond the clinical success of DBS, encompassing factors like postoperative neuropsychiatric symptoms, mood fluctuations, marital conflicts, incomplete understanding of DBS therapy, and limitations in social activities due to these conflicts.

Finally, the review highlights the potential for relational readjustment after surgery, with couples sometimes experiencing conflicts that necessitate a rapid redefinition of caregivers' roles. The transformation brought about by STN‐DBS may lead to newfound independence for the patient, altering the dynamics within the relationship.

In conclusion, this systematic review underscores caregiving's intricate and evolving nature in the context of STN‐DBS for PwP. It emphasizes the need for comprehensive support, education, and a nuanced understanding of the multifaceted challenges faced by CGs throughout this journey.

## AUTHOR CONTRIBUTIONS

**Francesco Cavallieri:** Conceptualization; methodology; data curation; formal analysis; writing – original draft; writing – review and editing. **Luca Ghirotto:** Conceptualization; methodology; data curation; writing – original draft; writing – review and editing. **Francesca Sireci:** Conceptualization; methodology; data curation; writing – original draft; writing – review and editing. **Margherita Parmeggiani:** Data curation; writing – review and editing; formal analysis. **Cristina Pedroni:** Data curation; formal analysis; writing – review and editing. **Felipe Andres Mardones:** Data curation; formal analysis; writing – review and editing. **Maria Chiara Bassi:** Methodology; data curation; formal analysis; writing – review and editing. **Valentina Fioravanti:** Data curation; formal analysis; writing – review and editing. **Valérie FRAIX:** Data curation; formal analysis; writing – review and editing. **Elena Moro:** Conceptualization; methodology; data curation; formal analysis; writing – original draft; writing – review and editing. **Franco Valzania:** Methodology; conceptualization; data curation; formal analysis; writing – original draft; writing – review and editing.

## CONFLICT OF INTEREST STATEMENT

None of the authors has any conflict of interest to disclose.

## Supporting information


FILE S1

FILE S2

FILE S3

FILE S4

## Data Availability

Data sharing is not applicable to this article as no new data were created or analyzed in this study.
