# Supplementary material for: Caregivers' burden and deep brain stimulation for Parkinson disease: A systematic review of qualitative studies
Source: Eur J Neurol. 2023 Nov 17;31(3):e16149. doi: 10.1111/ene.16149 (PMC11235895; doi:10.1111/ene.16149)
Supplement: Supplementary file 2 — FILE S2 [file ENE-31-e16149-s001.docx]

**Supplementary file 02 - Search strategies for each database**

**MEDLINE**

("Deep Brain Stimulation"[Mesh] OR deep brain stimulation OR subthalamic stimulation OR DBS OR bilateral high frequency stimulation) AND ("Parkinson Disease"[Mesh] OR parkinson) AND (Qualitative Research OR Grounded Theory OR Empirical Research OR Qualitat* OR Interview* OR Observation* OR Behavior Observation Techniques OR Narrat* OR Ethno* OR phenomenol*OR Focus Groups OR "Qualitative Research"[Mesh] OR "Focus Groups"[Mesh] AND "Grounded Theory"[Mesh] OR "Interviews as Topic"[Mesh] OR "Empirical Research"[Mesh] OR "Behavior Observation Techniques"[Mesh])

Filters: English; Field: Title/Abstract

**Embase**

'brain depth stimulation'/exp OR 'deep brain stimulation':ab,ti OR 'subthalamic stimulation':ab,ti OR dbs:ab,ti OR 'bilateral high frequency stimulation':ab,ti

AND

'parkinson disease'/exp OR parkison*:ab,ti

AND

'qualitative research'/exp OR 'focus group'/exp OR 'grounded theory'/exp OR 'empirical research'/exp OR 'behavioral observation'/exp OR 'qualitative research':ab,ti OR 'grounded theory':ab,ti OR 'empirical research':ab,ti OR qualitat*:ab,ti OR interview*:ab,ti OR observation*:ab,ti OR 'behavior observation techniques':ab,ti OR narrat*:ab,ti OR ethno*:ab,ti OR 'phenomenol*or focus groups':ab,ti

**Cinahl**

(MH "Deep Brain Stimulation") OR ( deep brain stimulation OR subthalamic stimulation OR DBS OR bilateral high frequency stimulation )

AND

(MH "Parkinson Disease") OR parkinson*

AND

(Qualitative Research OR Grounded Theory OR Empirical Research OR Qualitat* OR Interview* OR Observation* OR Behavior Observation Techniques OR Narrat* OR Ethno* OR phenomenol*OR Focus Groups)

**Cochrane**

deep brain stimulation OR subthalamic stimulation OR DBS OR bilateral high frequency stimulation in Title Abstract Keyword AND parkinson* in Title Abstract Keyword AND Qualitative Research OR Grounded Theory OR Empirical Research OR Qualitat* OR Interview* OR Observation* OR Behavior Observation Techniques OR Narrat* OR Ethno* OR phenomenol*OR Focus Groups in Title Abstract Keyword - (Word variations have been searched)

**PsycINFO**

exp Deep Brain Stimulation/ OR (deep brain stimulation or subthalamic stimulation or DBS or bilateral high frequency stimulation).ab,ti.

AND

exp Parkinson's Disease/ OR "parkinson*".ab,ti.

AND

qualitative methods/ or focus group/ or grounded theory/ or interpretative phenomenological analysis/ or narrative analysis/ or semi-structured interview/ or thematic analysis/

OR (Qualitative Research or Grounded Theory or Empirical Research or Qualitat* or Interview* or Observation* or Behavior Observation Techniques or Narrat* or Ethno* or phenomenol*OR Focus Groups).ab,ti.

**Scopus**

( TITLE-ABS-KEY ( deep  AND brain  AND stimulation  OR  subthalamic  AND stimulation  OR  dbs  OR  bilateral  AND high  AND frequency  AND stimulation )  AND  TITLE-ABS-KEY ( parkinson )  AND  TITLE-ABS-KEY ( qual*  OR  empirical  OR  focus  AND group  OR  narrative  OR  ethno*  OR  phenomenol*  OR  interview*  OR  observation* )
