# Supplementary material for: Caregivers' burden and deep brain stimulation for Parkinson disease: A systematic review of qualitative studies
Source: Eur J Neurol. 2023 Nov 17;31(3):e16149. doi: 10.1111/ene.16149 (PMC11235895; doi:10.1111/ene.16149)
Supplement: Supplementary file 3 — FILE S3 [file ENE-31-e16149-s003.docx]

**Supplementary file 03 - Critical Appraisal Results**

| **Articles** | **CASP score** | **Comments** | **Assessment** |
| --- | --- | --- | --- |
| Schüpbach et al., 2006 | 11 | Negative evaluations on method, ethics, discussion and privacy. | Low Quality |
| Gisquet et al., 2008 | 12 | Negative evaluations on method, presentation of results, ethical aspects and discussion. | Low |
| Haahr et al., 2013 | 27 | Negative judgment on method and discussion, everything else judged positively. | High |
| Lewis et al., 2015 | 27 | Negative evaluations on method, ethics and privacy, everything else judged positively. | Moderate To High |
| Liddle et al., 2018 | 26 | Positive evaluations on the method; negative evaluations on ethics and discussion. | Moderate To High |
| Liddle et al., 2019 | 26 | Positive evaluations on method and ethics. Negative evaluation of the discussion | High |
| Mosley et al., 2019 | 29 | Negative evaluations on method and discussion, everything else judged positively. | High |
| Thomson et al., 2020 | 29 | Positive evaluations on the method (except on the saturation of data), ethics and discussion. | High |
| Chacón Gámez et al., 2021 | 28 | Positive evaluations on the method (except on the saturation of data), ethics and discussion (except for transferability of findings). | High |
