# Supplementary material for: Caregivers' burden and deep brain stimulation for Parkinson disease: A systematic review of qualitative studies
Source: Eur J Neurol. 2023 Nov 17;31(3):e16149. doi: 10.1111/ene.16149 (PMC11235895; doi:10.1111/ene.16149)
Supplement: Supplementary file 4 — FILE S4 [file ENE-31-e16149-s002.docx]

**Supplementary file 04 - Categories, subcategories and participants' quotations**

| **Categories** | **Subcategories** | **Narratives** | **Frequency (articles)** |
| --- | --- | --- | --- |
| **Dealing with PD every day** | *Impacts* | “We have some great-grandchildren, and he loves them. They know when he starts making faces and starts going OFF, then his face ‘dies’ too and they leave him alone”^27^ | 3/9 |
|  |  | “He’d stay at home most days and watch TV all day. That was starting to bother me. Because that’s not who he is”^29^ |  |
|  |  | “About 15 years ago, she made a mistake with our boat and hit a buoy at high speed. Then the water police came (…) She was trembling all over her body and I have never seen her like this. That frightened me very much and since then, it happens whenever she gets upset (…) When she is tense, she has such uncontrolled movements. I first noticed in 2004 that when she is very excited or scared or something, she trembles”^28^ |  |
|  |  | “This has changed a lot, the disease. That is quite clear. Then, as you can see with the disease, where dyskinesia became more and more pronounced, comes the physiognomy change. I no longer knew my own wife by her face. It was so bad, the disfigurement that was caused by this illness that affects not only the movements but also the face”^28^ |  |
|  |  | “What I drew is what concerns me the most and touches me negatively, which is the effect as if he would have a mask on his face (…). I always have to think when I talk to him or when I get in touch with him whether there’s something going on. He just looks glassy-eyed. It’s not that there’s something wrong or that something has happened (…) It is somehow difficult for him to express that he is there for the other person in an empathic way. And the connection is somehow interrupted. But maybe that has to do with me too. I react very personally”^28^ |  |
|  |  | “It [PD] is autumn. Autumn. The leaves falling is not the end of life, but it’s the end of a life. It’s the end of the life before illness. So, it’s a time to mourn”^28^ |  |
|  |  | “Parkinson’s is like a cage for the person. The person is like in a cage for me and I can’t get into this cage”^28^ |  |
|  |  | “People with Parkinson’s, they lose their sense of reality over time. For example, when she cooks something, it goes in slow motion. When I say: ‘can’t be done faster?’. She says: ‘yes, I do work fast’. Or also with movements, when people with Parkinson’s walk, it’s centimeter by centimeter and when you ask them about it and say: ‘why don’t you take bigger steps? It’s better’, she says: ‘yes, I do take big steps’. It’s as if reality slowly disappears”^28^ |  |
|  |  | “That’s still a bit difficult for me now. Yes. In the past few years, I had the feeling that he was somehow isolating himself and he is like in a cage with his illness. Somehow, I have the feeling that I can’t get close to him anymore as if there would be like a Parkinson’s wall between us”^28^ |  |
|  |  | “He was so hyperactive, and I didn’t know if that was because he knew that he had a disease and he wanted to enjoy life (…) He was thinking more about himself, looking more for his own pleasure. He had no sense of time, and he was looking for his pleasure. That was his first concern, to think of himself”^28^ |  |
|  | *Limiting autonomy and socialization* | “Caroline stated: ‘We went to the party, but when we had finished dinner, he got cramps in his legs and went out and sat in the car. Nothing worked, and we went home. It is frustrating. You miss the rest of the party... dancing and such. I wouldn’t dream of sending him home in a cab and stay at the party’”^27^ |  |
|  |  | “Of course, I am not always at home, but usually we talk about how she feels, and if she is alright then I can go out”^27^ |  |
|  |  | “Mary reflected: ‘there are so many things he would like to do and knowing he wants to so much, I can’t bring myself to go, I cannot do that’”^27^ |  |
|  |  | “Christine said: ‘there has been loss of many things. Loss of the person you married, loss of abilities, loss of strength…I have experienced social isolation. Having to find a new identity, a new social life, a new way of living life’. She continued: ‘we have talked about what possibilities we have. How can we compensate for what we have lost?’”^27^ |  |
|  |  | “Peter said: ‘I have to think about being close by. If I go out, I bring the mobile phone’”^27^ |  |
|  |  | “Mary said: ‘everything that goes on in the evening, I have cut out’... and continues: ‘in daytime I do my own things, but I am never gone for a long time’”^27^ |  |
|  |  | “Susan said: ‘There must be room for me as well. When you say that, you can do a lot of things’”^27^ |  |
|  |  | “I would lie there – almost every night, just rubbing his legs, for the cramps to go away. And trying, just enough for him to be able to go to sleep. But sometimes that could take 45 minutes of me rubbing his legs, in the middle of the night”^29^ |  |
|  |  | “I think the disease itself is the devil on one side and the beautiful fairy on the other side. It is entirely day-dependent, time-dependent. Sometimes you could almost despair about the disease and other times, everything it’s quite normal and you can say to yourself that everyday life is actually quite normal. And then in the next half hour nothing works at all. What is also the problem is that we have to prepare every appointment very carefully”^28^ |  |
|  |  | “Our friends also noticed that he was so uninhibited and always restless. He always had to be stimulated and always had to listen to music. He also bought a lot of things like cars. He had a different behavior”^28^ |  |
|  |  | “For example, when we were at the table with the children, he ate a lot and very quickly, and then stood up and went to his computer. And that’s difficult with children, when you try to educate the children and say: wait until everyone has finished and then you can get up and leave the table. And he, he had no concept of parenting anymore”^28^ |  |
|  |  | “It’s just that for many couples it’s a big challenge. I see many who are trembling, and it’s easy, it wouldn’t work anymore if the partner didn’t have more patience. It’s tragic to see how that hurts you. There are so many different people. There are people who deal with it better and others who deal with it worse. You know, my son is washed up with it because he’s always had Parkinson’s around him. He experienced the grandfather yes. He came from school, he had to find the grandfather somewhere, he had to put him up again and bring him into the house. Then he experienced his father for 30 years. And now his partner and that is a lot for him at the moment and I think that often he just doesn’t have the energy anymore (…) She was very unwell before the operation and I just felt she had a nervous breakdown (…) I just notice that when she gets stressed, it comes through, and she needs another day [to recover]. And my son is allergic to it. He just almost can’t stand it. He didn’t want to be there today”   - Can I ask why?   “Something kind of broke.”   - In the relationship you mean?   “Unfortunately. That hurts me a lot” ^28^ |  |
|  |  | “Before the illness, she was really energetic and always had to do something, and now with the illness, I almost have to force her to go somewhere to the theatre or the cinema or somewhere. She has all the excuses she needs not to be around many people”^28^ |  |
|  | *Stepping aside for love* | “Mary said: ‘we have known each other for so many years and still love each other very much. And THAT’S the crux of the matter that we care so much for each other that we are positively in this together!’”^27^ |  |
|  |  | “Mary noted: ‘During the last months, his name has been written all over the calendar.’ She continued: ‘My life is our life...we live it together, and when he is well, then I am well’”^27^ |  |
|  |  | “Mary stated: ‘I sometimes cry when everything seems hopeless, but I seldom show him that I have been sad’”^27^ |  |
|  |  | “Victoria described a need to be strong: ‘I often feel I have to be the strong one. And often, he says to our friends that I am so strong. But deep down I am not. Sometimes I have the need to talk to somebody, as well’”^27^ |  |
|  |  | “Christine said: ‘I expect to be acknowledged as being part of this illness’ and continued: ‘It is good that the patient has to decide, but in our family, big decisions have always been something we are part of together’”^27^ |  |
|  |  | “I feel sometimes pressured because I make an appointment for her somewhere and calculate how long we need to get her ready and to be there. And then when we leave, it can happen that nothing works until we get to the station because it takes us a quarter of an hour to walk ten meters and then the train bye”^28^ |  |
|  |  | “What comes next? How much should I work? Should I take this job or better this one? (…) How will it go financially and how will it go later? How many years will it take before he needs care? What will I do then? Can I care for someone at all? I’m not the caring type of person. I noticed that a long time ago. Then I thought, oh my God, how do I do that then? It was always like a sword of Damocles hanging over you and you have to think, what’s next?”^28^ |  |
|  |  | “Something changed, I don’t know (…) Over time he became more of a patient and I became more of a caregiver, but I wouldn’t say from the beginning. It was over time, when more and more symptoms appeared (…) The illness has a strong influence on communication, because he often speaks very softly, because he speaks unclearly. Now, in the last few months, he is increasingly unable to express himself so well. The words don’t come out or he no longer knows what he wants to say, and his range of interests is simply more limited”^28^ |  |
|  |  | “In my opinion, too much is said about the sick person in our self-help group and not about oneself in the relatives’ group. So, I always bring that up there (…) I ask: ‘how are you doing’ and now they are starting to talk a bit more about themselves. (…) It helps to talk to people who have similar experiences. So, it’s also like not being alone. Talking about it with other people is always a bit difficult if they judge how it should be (…) But with those who have the same experiences, you only say I do it this way or I do it differently. And, um, yes, you also have to look after yourself””^28^ |  |
|  |  | “He had the impression that we were seeing something [PD symptoms], when it wasn’t at all (…) But he could see that, and he had the impression that everyone was paying attention to it. So, he didn’t want to talk about it too much, and I was the opposite. For me, to talk about it was a way of trivializing this illness. It was like saying I have the flu, yes, he has Parkinson’s, and then there you go. Then I talked a lot about it. It’s true, I pushed him to do it because it’s not keeping things inside that’s going to help”^28^ |  |
|  |  | “I do everything for my partner. Doctor talks, everything, and I’m there and I want to know what’s being done and yes. That’s everything for me. And that’s why, we manage, we do everything. It works”^28^ |  |
|  |  | “It is a double-edged sword. Dark clouds and clear bright sky (…) I have painted a small campfire here, and my wife’s tricycle, with the walker standing next to it. We live with these handicaps, with these difficulties, but we always enjoy the sea and the view. We see a horizon (…) The ship disappears in the horizon to unknown places. We don’t see exactly where we are going but we are inside this ship and hope that it leads to a good destination towards the sun. The flowers at the beach indicate that we are also having a good time. We have experienced a lot of beautiful things, we were lying in the sun, here the chairs have become empty now, but we are still here (…). Seen from my point of view, it is a hopeful picture, which nevertheless has the shadows of everyday life, and it shows that there are also dark sides, stony paths, or you can be alone sometimes and still be together as a couple as long as it is possible. The fire is still burning, maybe a small one (…) Even our living together, our intimate life, that hasn’t been extinguished”^28^ |  |
|  |  | And yes, how should I put it, um, because of the illness she has also become more selfish. So, she comes first and then again and then maybe the others. Sometimes I’ve also said, I’m not a domiciliary care provider. You pay him and you can give him orders, but I don’t get paid”^28^ |  |
| **Facing life changes with STN-DBS** | *Feeling unprepared* | “Had they said to me he may have a change of personality, then I could have said well this has happened, and got on to it sooner. From my point of view, I’ve had to learn the hard way about the side effects”^32^ | 8/9 |
|  |  | “I have a sense that while there’s a lot of experience around, there’s not a lot of firm, really solid knowledge about what happens once we start tweaking [adjusting the DBS]. It’s very - there’s a real trial and error aspect to it, which I probably thought would be less the case - that it would be more known, more rigorous if I can put it that way. I understand that it’s a fairly new technology, so we’re part of that developing of that rigour”^32^ |  |
|  |  | “We haven’t done any counselling at all, and I think we need to. As a spouse, you need to be prepared that these things can happen, and that husbands or partners can turn feral [wild] and not to - we were told not to take it to heart - whatever is said is said out of - they can’t help it. But in saying that, that’s kind of not enough. You still hold - I mean, I do - I still hold on to things that were said and things that were done because it’s ultimately affected our relationship. That’s something that I have to move on from but it’s really difficult”^32^ |  |
|  |  | “It’s a bit like childbirth [laughs]. It’s kind of like no matter how much preparation you do, it’s just something you’ve got to experience yourself... Truly we didn’t think that - we sort of just hoped we’d skate through without having those kinds of experiences”^32^ |  |
|  |  | “Definitely the behaviour side of it… because that was really quite scary… he would just go! Whatever he’d thought he’d just go and do it… We had no understanding that could just be changed by changing the controls… So, I think they need to tell people that, because if it had gone on and just let him do whatever… Well, I wondered why—every time you go to a neurologist appointment, they’d ask you, ‘oh, is there any change in behaviour?’… gambling, sort of, alcohol-type behavior… I’m going ‘no’… then when that happened… ‘oh, now I know why you ask that all the time!’”^27^ |  |
|  |  | “When you read up on the deep brain stimulation, it says it can actually make them [ICDs] worse. I’m thinking, oh my God, how much worse can it get?!”^27^ |  |
|  |  | “I think both people need to be well informed. I think that’s important and even other family members and or friends. I really went out of my way. Dianne doesn’t even know this, but people would send me stuff about Parkinson’s, and I’d likewise send it out to other people so that everyone had a bit of an understanding”^31^ |  |
|  |  | “Does the medicine work right away? Doesn’t it work yet? Uh, a lot of things are happening at the same time. It’s very difficult and each person is very different”^28^ |  |
|  |  | “I have to say, I expected more. I thought after the brain operation everything would be fine, yes, fine, the hope was there, now everything will be fine again. If I am stimulated every day afterwards it’ll be like before. But that wasn’t the case. That was only at the beginning. The shared joy (…) It’s only possible to adjust it so that it is optimal (…) And that ideal point, was not always ideal. At the beginning, they had to change it a bit up, a bit down, and then it is found wasn’t the best result. Ah, it’s the best possible, but not what we had hoped for”^28^ |  |
|  |  | “And he had it again a fortnight ago. So, depression-like episodes (…) No one told me that could happen (…) He wasn’t told either. We talked about it recently. I told him, why don’t you ask that in the next consultation? I told him that when he has an examination in hospital, then he should ask whether this side effect is possible”^28^ |  |
|  | *Experiencing patients’ behavioral changes with concern and fear* | “Hmm, hmm. Yes, well, he gets us into trouble, or he could get us into trouble, with things that might cause us problems”^34^ |  |
|  |  | “He is not like he was before. He gets annoyed over the smallest things. He is almost aggressive towards me sometimes. I don’t understand”^30^ |  |
|  |  | “Seriously hyper, that really concerned me”^31^ |  |
|  |  | “Family members were more likely to describe some concerns about the way in which the changed symptoms related to changes in daily life. Some expressed concern that the person with PD was not doing more and embracing independence, while others were concerned about the magnitude and rapidity of the changes experienced.”^31^ |  |
|  |  | “I’d wake up, and I’d hear this noise, and he’d be scrubbing the skirting boards”^29^ |  |
|  |  | “Richard explained: ‘I can tell right away by looking at her face. Then I try to help her, by holding her hand or grabbing her by the arm and continued... suddenly she says: ‘I am going OFF’ and then she is tripping away. It happens so quickly, and you can see it in her face”^27^ |  |
|  |  | “I wouldn’t want to see any kind of change that was too dramatic… if he was to become really outgoing or really inward or really selfish or—I wouldn’t want any of those in exchange for his physical capabilities”^27^ |  |
|  |  | “He talked incessantly, non-stop, and just kept swapping from topic to topic to topic... He’d just ring people up and go, ‘oh, I’ll pick you up in 10 minutes’... but the neurologist just changed channels or whatever, and that disappeared… Apparently, he was on a high with it. It was like being on drugs and stuff””^27^ |  |
|  |  | “My biggest fear... I can cope with absolutely anything. If he’s quadriplegic, it’s fine, I can deal with that, but I can’t deal with – the psychiatric changes, it scares me too much. How he behaved, how he was when he was back there, I can’t do that again”^32^ |  |
|  |  | “This illness is something different. Everything else in your life, you work harder, you tough it out, and whether it’s a problem in a relationship or whatever, you work through it. This shit, it’s all in someone else’s hands. The most helpless feeling you’ll ever have. It really is”^32^ |  |
|  |  | “If he has to speak for a long time, usually his voice will diminish, he won’t be able to (…) He enjoys going to restaurants, eating, something he didn’t enjoy before. But on the other hand, he can’t express himself when he is in society and has to speak when there are a lot of people (…) So that’s one of the disadvantages”^28^ |  |
|  |  | “He really had a personality change for a short period of time and also a maniac phase. He was completely different for a while.”   - What do you mean by manic phase?   “Yes, after the operation he was really changed in his manner, that he for example/that he complimented me or hugged me when greeting me, as he never did before (…) He bought an expensive watch and booked holidays, big holidays, without discussing it with my mother. And also wanted to write a book. Yes, things like that”^28^ |  |
|  | *Struggling to find an explanation* | “Susan said: ‘We have been told that his speech can be affected, but you get frustrated anyway and find it hard to understand... because I am not ill’”^27^ |  |
|  |  | “If she’d have seen a video of herself, she’d have been surprised. In her mind, she thinks that she was fine, and she still believes that she was fine. But you understand from her point too because of where that wire was, she was high, for want of a better term, and feeling like a million bucks... that she’s like superman. Almost like someone on drugs, but didn’t believe that anything she was doing was wrong... It didn’t get better. It just escalated. Every time they turned the unit, the voltage up she went up a level... You can’t believe that a little bit of voltage would shift someone from there to there in that little bit of time”^32^ |  |
|  |  | “I’ve seen with this that people can change pretty quick just from a wire. Same person, same mind, or same brain, just shift a bit of voltage somewhere and a different person ... But I don’t see why I should condone bad behaviour. Whether you’re crook [ill] or not, bad behaviour is bad behaviour”^32^ |  |
|  |  | “To some extent, I think that they’re probably - at least in [person with PD’s] case kind of unique to him. They’re not alien to what he used to be. They’re exaggerations of how he would once have behaved anyway... it’s just like everything with the knobs turned up, or the volume turned up... we’re back to the 20-25-year-old personality that’s not very nuanced and not all that willing to compromise”^32^ |  |
|  |  | “I don’t know what’s [the person with PD] anymore and what’s the DBS. I don’t know if he’s changed as a person or if a lot of it is the DBS. It’s a hard one to answer because I’m confused in my head of what’s real and what’s not real anymore”^32^ |  |
|  |  | “She was always a bit feisty beforehand, but now… She does get very defensive very quickly… It could be a mixture I think, of the DBS and muddling with her brain, and the fact that her Parkinson’s has progressed, and the [cancer-related] operation. Whether [the cognitive changes] were just marred by the movement prior to and you concentrate just on one thing and forget about the others...because we concentrated so much on the movement and trying to help with that.”^27^ |  |
| **Rebuilding the role of CG/partner after STN-DBS** | *Learning dealing with changes* | “There’s going to be more stressful times, I’m sure”^31^ | 9/9 |
|  |  | “This man, this personality changes he’s gone through, it’s crazy. It’s not—he’s not the man I married. He’s definitely not the man I married. He’s changed so much. If that’s just part and parcel of Parkinson’s, I guess?”^27^ |  |
|  |  | “Caregivers spoke of their partner ‘no longer being the person I married”^32^ |  |
|  |  | “It was actually quite scary, his behaviour and stuff… was quite erratic... [I’m] thinking is this what the future basically is with this person? Have we done the right thing? Because he was a little bit awkward to live with for a little while there”^27^ |  |
|  |  | “We call him the Energizer Bunny, and when the friends walk in, they’ll say to him, are we switched on today, or switched up, because he’s just got this energy. Then when you turn him down... in the afternoon, he’d have to have a little nap. Well, he doesn’t like that. He likes to have this Energizer Bunny energy. Since he’s had a taste of it, he really likes it. It’s almost like an addiction, actually... to me, it’s almost like control”^32^ |  |
|  |  | “… and described a change in the spousal role to that of ‘parent’, ‘flatmate’, ‘nurse’ or even ‘sexual object’. Those with younger children struggled to explain behavioural changes to their offspring. Caregivers were troubled by verbal disinhibition and irritability, rating this as more burdensome than motor symptoms”^32^ |  |
|  |  | “Ever since the operation, I feel lost. Before, when he was sick, we were a perfect couple. Now, he wants to live the life of a young man, go out, meet new people, all of that is intolerable! I would rather he be like he was before, always nice and docile!”^33^ |  |
|  |  | “He didn’t want me looking after him and was calling me controlling, whereas normally it was - as I said, we were just a team. I don’t call it controlling. I call it helping… it just triggered some… dark side”^32^ |  |
|  |  | “I was used to doing a lot of the jobs myself [before surgery], then he’d come in, and he’d start doing things without any sort of consultation. The first six months were pretty rocky”^31^ |  |
|  |  | “I was used to doing a lot of the jobs myself, and then he’d come in and start doing things without any sort of consultation’”^29^ |  |
|  |  | - How would you rate your present quality of life? On the same scale, from one-worst quality of life to ten-better quality of life, where would you see yourself?   “Ah well, around six (…).”   - What do you miss at the moment?   “The stress, really. I have to get used to that, the organization of taking the tablets is less now, and I have to calm myself down. I know that […]”^34^ |  |
|  |  | “We went to (country) for four days on holiday, because I insisted… it was tough, and I was curious how it would go, but it was ok... we could drive there... not further away from home than we could come home quickly. So... we had to try”^27^ |  |
|  |  | “Next Friday we are going to our neighbors’ party, and I really look forward to it. I expect that we can stay there till the end”^27^ |  |
|  |  | “Any work colleagues or friends of his [say], ‘it’s great to see the old [patient name] back, we were really worried there for a little while’… now I feel like I’m not totally, you know, he’s back, so I’m not alone again. So that’s good”^27^ |  |
|  |  | “It’s better, but you still live next to a sick person, and you sleep next to a sick person”^28^ |  |
|  | *Renaissance* | “I feel like I have got a new husband… we are much closer… it is almost like being in love again”^27^ |  |
|  |  | “Altogether we are happy with the treatment. I am probably the one who is most happy. It is a paradox, but I think so. In some ways I have got my husband back”^27^ |  |
|  |  | “When I saw him, it was just like it was almost a miracle because he had the typical frozen face. And I looked at him in the chair, and his face was alive again. Unbelievable”^31^ |  |
|  |  | “We love each other very much and that is, of course, alpha and omega’”^27^ |  |
|  |  | “I’ve got my wife back’”^27^ |  |
|  |  | “When she had the Parkinson’s—she used to worry a lot. She used to be really down. Now she’s sort of the old [spouse] I know. She laughs at my jokes, even how corny they are… yeah so, no, she’s a much happier person”^27^ |  |
|  |  | “If he didn’t have the DBS, then he would have been excluded from a lot of it”^29^ |  |
|  |  | “After DBS Mary said: ‘…we don’t feel disabled as we did before DBS’”^27^ |  |
|  |  | “Caroline reflected: ‘you wonder….is it really true? …but it is. I can go, and I don’t have to arrange for anybody to check on him. He can help himself. It is a bit weird and really really nice’”^27^ |  |
|  |  | “Richard said: ‘…if we want to go to town, then we just go. We don’t have to wait for any medication to work’ and he continued: ‘Things don’t have to be planned. We can decide to go whenever. We don’t have to wait until the ‘right time .’Or worry that she will go OFF’”^27^ |  |
|  |  | “Christine said: ‘I am very alert that he doesn’t get too comfortable… such as he asks me to get him things and I tell him to get up and get them himself,’ and Helen said: ‘Now he has to take care of his medicine himself. I used to do that, but I don’t anymore… I keep an eye on him, but he doesn’t know that’”^27^ |  |
|  |  | “DBS ‘gave me a peace of mind for me that he can dress himself, shower himself and cook for himself’”^31^ |  |
|  |  | “Since then, she can use her hand completely again. She doesn’t tremble. She can do different things by herself again. Before I had to cut the meat and everything for her, and today everything is back to normal”^28^ |  |
|  |  | “And then after I drew spring, because the stimulation (DBS) is hope, renewal, and then it’s life that blooms again”^28^ |  |
|  |  | “He is more at ease when speaking (…) He can stay standing or go with them [the neighbors] to see something. Whereas before he withdrew himself a little when he had so much dyskinesia. I do think that it’s better now for the contact and the neighborhood network”^28^ |  |
|  |  | “Now it’s better after the deep brain stimulation. It is really better (…) The speaking part that is very important for me, that you can exchange and talk to each other. Of course, that became less. Parkinson’s patients also speak less, of course. That was also a huge problem for me. I like to talk about everything. I want to exchange ideas. That was no longer possible. That is better now”. “Now here I have the hope, partly after the deep brain stimulation, that even if it is a bit worse in between, it will get better again”^28^ |  |
|  |  | “No, nothing bothers me about her, even that she has such a device above her chest that you can see and feel, that doesn’t bother me. (…) That belongs to my wife. Exactly. It’s not a foreign body from my point of view. I don’t perceive her as my wife, who has electronics in her brain. I just don’t think about it at all”^28^ |  |
|  | *Estrangement* | “Susan reflected on: ‘Suddenly there is nothing there for us to look forward to. We have to deal with the situation as it is’”^27^ |  |
|  |  | “Susan said: ‘I am really tired. I really am. A lot of things have happened, and as they [doctors] tell us, the illness is still progressing’”^27^ |  |
|  |  | “Not really. He thinks it’s his business (referring to gambling via telephone) and that he has the responsibility, and that I have nothing to do with it. I tell him that I do have something to do with it, because if he does something like that and they clear out our bank account… then I’ve got as much of a problem as he has”^34^ |  |
|  |  | “C. How should I say this… he is very lazy. No energy at all, nothing. […]. I go to work in the mornings, and when I get home, I have to do all the housework. He does no hoovering, no dusting, nothing at all. Do you see? He gets up after me in the mornings, but he doesn’t open the blinds or lay the table. Nothing, even though these are only small chores.”   - And when you ask him about this, is he at all reasonable about it?   “C. Well, yes, he says, ‘I’ll have to change something.’ But nothing happens, and he doesn’t change anything. My view is that, strictly speaking, if I didn’t swallow everything, then we would get into a fight every day–and that wouldn’t be a life worth living anymore”^34^ |  |
|  |  | “Peter said: ‘You have become more tied to the home. You cannot just go without anyone knowing where you are’”^27^ |  |
|  |  | “I gave of myself all these years while he was sick, avoiding saying or doing anything that might be hurtful, but now I can’t stand it anymore; he makes no effort, doesn’t budge, and waits for me to do everything”^33^ |  |
|  |  | “It is noticeable today that everything has become a little slower (…) The asking back and forth, that has increased. In the past she cooked, I had no problem, I ate what she made. Today I have to ask her, what would you like for dinner today? That has become our daily routine, three times a day, or, in the morning, I say, what would you like, bread, everything, at noon and in the evening. Yes, that has become my task, to think a bit more for my wife as well.”   - More after the intervention than before?   “Yes, before I didn’t have to think for my wife anything. She organized everything herself and was independent in every way. She managed the household, but today we have to share everything”^28^ |  |
|  |  | “It simply means that life in society is different. It means that he is often in a chair at home, uh, because of fatigue. Fatigue and then walking, eh walking, it has decreased a lot too”^28^ |  |
